# Health insurance ownership among female workers in Indonesia: does socioeconomic status matter?

**DOI:** 10.1186/s12889-022-14189-3

**Published:** 2022-09-22

**Authors:** Agung Dwi Laksono, Wahyu Pudji Nugraheni, Nikmatur Rohmah, Ratna Dwi Wulandari

**Affiliations:** 1 National Research and Innovation Agency Republic of Indonesia, Jakarta, Indonesia; 2 Faculty of Health Science, Muhammadiyah University of Jember, East Java, Indonesia; 3 Department of Administration and Health Policy, Faculty of Public Health, Universitas Airlangga, Surabaya, Indonesia

**Keywords:** Health insurance, National health insurance, Health policy, Population survey, Public health

## Abstract

**Background:**

Female workers are vulnerable groups in the Indonesian context, and female workers must be responsible for domestic problems and earn a living. The study aimed to analyze the role of socioeconomic on health insurance ownership among female workers in Indonesia.

**Methods:**

The study population was all female workers in Indonesia. This cross-sectional study involved 7,943 respondents. The study analyzed health insurance ownership as an outcome variable and socioeconomic status as an exposure variable. The study also involved five control variables: residence, age, marital, education, and occupation. The research used multinomial logistic regression in the final step.

**Results:**

The results show the poorest female workers have a possibility of 0.735 times more than the richest to have NHI (AOR 0.733; 95% CI 0.733–0.737). The poorer female workers have 0.939 times less likely than the richest to have NHI (AOR 0.939; 95% CI 0.937–0.942). Female workers with middle socioeconomic status are possibly 0.833 times less than the richest to have NHI (AOR 0.833; 95% 0.831–0.835). Moreover, the richer female workers have 1.028 times more likely than the richest to have NHI (AOR 1.028; 95% CI 1.025–1.030). Moreover, all socioeconomic statuses have a lower possibility than the richest of having other health insurance.

**Conclusions:**

The study concluded that socioeconomic has a role in health insurance ownership among female workers in Indonesia.

## Introduction

Women in the Indonesian context are a vulnerable group. Women who work have a dual role: taking care of the household and working to earn a living. Female workers today face conflicting demands from both work and life fields, and women find it challenging to manage and balance workplace pressures and household tasks. Balancing work and household is critical for female workers [1]. Female workers in Indonesia tend to work in the tertiary sector (trade, hotels, restaurants, transportation, finance, communications, leasing, and services) rather than the primary sector (agriculture, mining, and quarrying) [2]. Some barriers to female workers in Indonesia include career disruption due to childbirth and childcare responsibilities, lack of education and skills, low wages, unsupported gender-based regulations, and low support for gender equality [3, 4]. Another study mentions a weakness in married female workers. Married female workers tend to be less patient in the service process; they want to handle complaints quickly and as soon as possible [5]. The double burden of female workers is related to the physical workload of caring for the house and working as laborers and the psychological responsibility of ridicule for not doing a good job [6, 7]. This situation makes female workers vulnerable to physical and mental conditions; compared to women who only play homemakers, female workers are more prone to illness [6–8]. Once they are sick, and out of work, there is a lost income opportunity. The economic resilience of female workers is really tested when they are sick. For this reason, having health insurance is very important for female workers.

Indonesia has committed to implementing national health insurance (NHI), including supporting the poor [9]. Everyone in Indonesia has the same rights in obtaining access to health sector resources and health services that are safe, quality, and affordable. On the other hand, everyone in Indonesia also must participate in the social health insurance program. To reduce the risk of people bearing health costs out of pocket, in amounts that are difficult to predict and sometimes require very high prices, a guarantee in the form of health insurance is needed. Thus, the health financing is borne jointly by all participants, not to burden each person. But health insurance alone is not enough; we need social health insurance or NHI. Social health insurance applies the principles of cost and quality control, and the situation means that participants can get adequate quality services at reasonable and controlled prices. Social health insurance guarantees certainty of financing for sustainable health services, and we can use it throughout Indonesia. Therefore, the Government of Indonesia has released NHI to address inequality in access and quality of health services, ensuring that all citizens, especially the poor and near-poor, can access quality care without facing financial difficulties [10].

The reported coverage of NHI participation based on a secondary analysis of four nationally representative quantitative data in 2018 states: that self-reported NHI registration (60.6%) is about 10% lower than insurance reports (71.1%). Insurance coverage is highest in poor areas [11]. Based on the integrated monitoring and evaluation system, the Indonesian National Social Security Council reports that the national NHI membership is 86.96%, the Non-Contribution Assistance Recipient (NAR) is 40.4%, and the Contribution Assistance Recipients (CAR) is 59.6%. The government reports participation based on membership segment up to December 2021: workers receiving 25.5% wages; non-wage recipients 13.1%; non-workers 1.9%; central CAR 42.4%; and regional CAR 17.1%. The profiles of wage recipient workers participants up to December 2021 are as follows: civil servants 20.8%), Indonesian national armed forces/republic of Indonesia police/civil servants ministry of defense 4.6%; non-civil servant government employees 5.5%; and private/state-owned enterprises/other employees 68.9%. NHI participation by gender shows almost equal; data reported up to December 2021, 49.5% female and 50.5% male [12]. This data is balanced with the Indonesian population until December 31, 2021; the number of men in Indonesia is more than women. Indonesia's people of the male sex are recorded at 138.3 million people (50.5%) and 135.57 million women (49.5%) [13].

Studies in West Java report that per capita household expenditure, number of unproductive household members, household members, residence status, Gross Domestic Product (GDP) per capita, and agricultural contribution to GDP affect women workers [14]. In comparison, a study in East Java reported that heads of households have a higher probability of working than non-heads of homes. Each increase in family members will reduce women's opportunities to work [15]. In 31 of 34 provinces in Indonesia, women receive fewer wages than men; this will significantly hamper the role of women in poverty alleviation because women workers will find it difficult to achieve financial independence and economic independence [16]. Studies in Belgium and China report that workplace poverty has become chronic, especially among women workers in low-quality jobs [17]. Limited leisure time (time poverty) due to the burden of caregiving can prevent women from fully engaging in the formal economy, limiting productivity and economic growth and encouraging self-neglect [4]. The vulnerability experienced by female workers from low-income families is motivated by weak economic conditions; their husbands generally work modestly and odd jobs. The income earned is insufficient for the family's needs [6].

Women work in very diverse economic sectors. Women work longer hours than men. Women still carry out household responsibilities regardless of employment status [4]. Children of non-professional women workers are at risk of health problems. Children whose mothers worked in agriculture and manual work had a higher chance of experiencing stunting compared to those whose mothers worked in professional jobs [18, 19]. Studies in Peru show that 82.1% of working mothers work for their family members, do not receive remuneration, and are not paid. Children of mothers who do unpaid work are at higher risk of stunting [20]. The chance of stunting in Indonesia increases significantly among children living in households with three or more children under five. The odds also increase significantly with a decrease in the household wealth index [21]. Women's financial autonomy is one of the factors associated with reducing the likelihood of children becoming stunted [22, 23]. In addition to causing time poverty for themselves, female workers also impact their children's health.

Women currently comprise almost half of the national workforce. Female workers make up 70% of mothers with children under 18. The average income for women is 81% of the payment for men [24]. Moreover, employment-based health financing schemes tend to favor men and undermine progress towards universal health coverage. Achieving universal health coverage requires progress financing systems that ensure women access adequate, appropriate, affordable, quality health care. The situation jeopardized access to health care when working with their job requirements, as women face more jobs and transitions throughout their lives, including for reproduction and unpaid work [25, 26]. Female workers in low socioeconomic conditions (poor and near-poor) will have limitations in buying health insurance. Female workers need economic security covering health problems, insurance protection, and paid sick leave [24]. Thus, policymakers must ensure accessibility reduces barriers for women workers to health services. Policymakers must be able to encourage increased NHI participation for women workers. Based on the background, the study aims to analyze the socioeconomic role of ownership of health insurance for female workers in Indonesia.

## Materials and methods

### Study design and data source

The study design was a cross-sectional study. The study used secondary data from a previous study ("Abilities and Willingness to Pay, Fee, and Participant Satisfaction in implementing National Health Insurance in Indonesia in 2019"). The survey conducted data collection on respondents aged ≥ 18 years. This survey aims to determine the ability and willingness to pay contributions, cost reductions, and participants' satisfaction in implementing NHI. The survey was a national-scale survey conducted by the Ministry of Health of the Republic of Indonesia. Through stratification and multistage random sampling. The survey collected 47,644 respondents [27].

Meanwhile, this study population was all female workers in Indonesia. By filtering by gender (female) and employment status (employed), the study described 7,943 respondents. Sample distribution by province can be seen in Table 1—moreover, the distribution of the variables is displayed in Table 2.

**Table 1:** Sample distribution by province

Province	n	%
Aceh	104	1.3
Sumatera Utara	387	4.9
Sumatera Barat	134	1.7
Riau	106	1.3
Jambi	48	.6
Sumatera Selatan	221	2.8
Bengkulu	53	.7
Lampung	180	2.3
Kep.Bangka Belitung	37	.5
Kepulauan Riau	37	.5
DKI Jakarta	328	4.1
Jawa Barat	1231	15.5
Jawa Tengah	1404	17.7
DI Yogyakarta	131	1.6
Jawa Timur	1458	18.4
Banten	316	4.0
Bali	185	2.3
Nusa Tenggara Barat	197	2.5
Nusa Tenggara Timur	208	2.6
Kalimantan Barat	155	2.0
Kalimantan Tengah	89	1.1
Kalimantan Selatan	139	1.7
Kalimantan Timur	67	.8
Kalimantan Utara	15	.2
Sulawesi Utara	65	.8
Sulawesi Tengah	83	1.0
Sulawesi Selatan	261	3.3
Sulawesi Tenggara	56	.7
Gorontalo	28	.4
Sulawesi Barat	24	.3
Maluku	53	.7
Maluku Utara	19	.2
Papua Barat	29	.4
Papua	95	1.2
Total	7943	100.0

**Table 2:** Variable distribution

Variable	n	%
Health Insurance Ownership		
Uninsured	2030	25.6
NHI	5696	71.7
Other	217	2.7
Wealth status		
Poorest	1950	24.5
Poorer	1529	19.2
Middle	1407	17.7
Richer	1404	17.7
Richest	1653	20.8
Type of Residence		
Urban	2366	29.8
Rural	5577	70.2
Age group		
0—17	87	1.1
18—64	7296	91.9
> 64	560	7.1
Marital status		
Never married	1212	15.3
Married	5384	67.8
Divorced/Widowed	1347	17.0
Education Level		
No education	509	6.4
Primary	4374	55.1
Secondary	2105	26.5
Higher	955	12.0
Occupation type		
Civil servant/Police/Army	477	6.0
Private sector	1003	12.6
Entrepreneur	2376	29.9
Farmer/Fisherman/Labor	2749	34.6
Others	1338	16.8

### Variables

The study employed health insurance ownership as the outcome variable. There are three groups of health insurance ownership: uninsured, NHI, and other health insurance. The survey only asked about the primary type of insurance owned. Meanwhile, the study used socioeconomic as an exposure variable.

The survey employed the wealth index formula to determine socioeconomic status. The poll formulated the wealth index based on a weighted average of a family's overall spending. Meanwhile, the wealth index was calculated based on primary household expenditures such as health insurance, food, accommodation, and other items. Furthermore, the poll separated the income index by quintile into five categories: the poorest, poorer, middle, richer, and the richest [28–30].

As control variables, the analysis used five variables. The five variables were the type of residence, age group, marital status, education level, and occupation type. There were two types of residence available: urban and rural. The study split the age group into three classes in the sample: ≤ 17, 18—64, and ≥ 65. Meanwhile, the study divided marital status into three categories: never married, married, and divorced/widowed. There were four levels of education: no education, primary, secondary, and higher education. Moreover, there are five occupation types: civil servant/police/army, private sector, entrepreneur, farmer/fisherman/ labor, and others.

### Data analysis

The research employed the Chi-Square test to make a bivariate comparison in the first stage. Next, the researchers used a collinearity test to ensure no intense correlation between independent variables in the resulting regression model. In the final step, the study employed a multinomial logistic regression. The researchers conducted the last step to explore the multivariable relationship between all independent variables and health insurance ownership as the outcome variable. The researchers employed the IBM SPSS 26 software for the duration of the statistical analysis.

### Ethical approval

The study used secondary data from the "Abilities and Willingness to Pay, Fee, and Participant Satisfaction in implementing National Health Insurance in Indonesia in 2019" survey, so it does not require ethical approval. The National Ethics Committee has approved the "Abilities and Willingness to Pay, Fee, and Participant Satisfaction in implementing National Health Insurance in Indonesia in 2019" survey's ethical clearance (Number: LB.0201/2/KE.340/2019). The poll deleted all the identities of respondents from the dataset.

## Results

### Descriptive results

The study results show that the socioeconomic status distribution among female workers in Indonesia is: the poorest at 25.8%, poorer at 19.6%, middle at 17.3%, richer at 17.4%, and the richest at 19.9. Meanwhile, the distribution is uninsured 26.0%, NHI 71.3%, and other insurance 2.7% based on health insurance ownership.

Table 3 shows the descriptive statistic of the respondents' socioeconomic and individual characteristics. The poorest dominated the uninsured and NHI categories based on health insurance ownership; the richest led the other class.

**Table 3:** Descriptive statistic of socioeconomic and individual characteristics of respondents in Indonesia, 2019 (*n* = 7,943)

Characteristics	Socioeconomic					p-value
	**Poorest** **(*n* = 1,950)**	**Poorer** **(*n* = 1,529)**	**Middle** **(*n* = 1,407)**	**Richer** **(*n* = 1,404)**	**Richest** **(*n* = 1,653)**	
Health insurance ownership						< 0.001
Uninsured	32.3%	20.1%	18.5%	14.8%	14.4%	
NHI	23.8%	19.5%	17.0%	18.4%	21.3%	
Others	13.5%	17.9%	11.5%	19.0%	38.1%	
Type of residence						
Urban	13.8%	13.3%	17.2%	22.7%	33.0%	
Rural	30.1%	21.9%	17.3%	15.5%	15.2%	
Age group						< 0.001
≤ 17	24.4%	26.2%	13.9%	19.4%	16.0%	
18—64	23.4%	19.8%	17.7%	18.0%	21.0%	
≥ 65	57.6%	15.2%	11.4%	9.1%	6.8%	
Marital status						< 0.001
Never married	21.4%	16.8%	16.4%	21.5%	23.9%	
Married	21.2%	20.2%	18.6%	18.2%	21.8%	
Divorced/Widowed	48.3%	19.5%	12.6%	10.6%	9.0%	
Education level						< 0.001
No education	49.2%	20.9%	12.1%	11.0%	6.8%	
Primary	32.2%	22.7%	18.9%	14.6%	11.5%	
Secondary	14.4%	16.1%	17.6%	24.4%	27.5%	
Higher	6.1%	11.1%	11.0%	19.3%	52.5%	
Occupation Type						
Civil servant/Police/Army	7.8%	11.2%	11.4%	19.0%	50.6%	
Private sector	11.6%	13.3%	15.4%	24.5%	35.2%	
Entrepreneur	16.8%	17.6%	18.9%	21.7%	24.9%	< 0.001
Farmer/Fisherman/Labor	42.6%	23.5%	16.4%	10.9%	6.6%	
Others	21.8%	22.1%	19.6%	18.4%	18.2%	

Regarding the type of residence, the richest occupied the urban area, and on the contrary, the poorest led in the rural area. According to the age group, the poorer dominated in ≤ 17 groups, and the poorest dominated in 18–64 and ≥ 65 groups.

The richest occupied in never married and married categories based on marital status, and meanwhile, the poorest led in the divorced/widowed category. The poorest dominate in no education and primary levels based on education level, while the wealthiest dominate in secondary and higher levels.

Table 3 shows, based on occupation type, the richest led in civil servant/police/army, private sector, and entrepreneur types. Moreover, the poorest conducted in farmer/fisherman/labor and other classes.

### Multivariate regression analysis

The collinearity test shows no significant relationship between the independent variables. Furthermore, the tolerance value for all variables is more potent than 0.10, and the variance inflation factor (VIF) value for all variables is less than 10.00. According to the findings, the regression model exhibited no multicollinearity, implying that the test's decision-making foundation was sound.

Table 4 shows the multinomial logistic regression of health insurance ownership among female workers in Indonesia. The poorest female workers have a possibility of 0.735 times less than the richest to have NHI (AOR 0.733; 95% CI 0.733–0.737). The poorer female workers have 0.939 times less likely than the richest to have NHI (AOR 0.939; 95% CI 0.937–0.942). Female workers with middle socioeconomic status are possibly 0.833 times less than the richest to have NHI (AOR 0.833; 95% 0.831–0.835). Moreover, the richer female workers have 1.028 times more likely than the richest to have NHI (AOR 1.028; 95% CI 1.025–1.030).

**Table 4:** The result of multinomial logistic regression of health insurance ownership among female workers in Indonesia, 2019 (*n* = 7,943)

Predictors	NHI			Others		
	**AOR**	**95% CI**		**AOR**	**95% CI**	
		**Lower Bound**	**Upper Bound**		**Lower Bound**	**Upper Bound**
Socioeconomic: Poorest	^*^0.735	0.733	0.737	^*^0.254	0.252	0.256
Socioeconomic: Poorer	^*^0.939	0.937	0.942	^*^0.495	0.492	0.498
Socioeconomic: Middle	^*^0.833	0.831	0.835	^*^0.320	0.317	0.322
Socioeconomic: Richer	^*^1.028	1.025	1.030	^*^0.583	0.579	0.587
Socioeconomic: Richest	-	-	-	-	-	-
Residence: Urban	^*^1.750	1.747	1.754	^*^1.658	1.650	1.666
Residence: Rural	-	-	-	-	-	-
Age: ≤ 17	^*^0.250	0.248	0.252	^*^2.406	2.353	2.460
Age: 18—64	^*^0.809	0.806	0.812	^*^1.358	1.342	1.374
Age: ≥ 65	-	-	-	-	-	-
Marital: Never married	^*^0.887	0.884	0.889	^*^0.229	0.227	0.231
Marital: Married	0.998	0.996	1.000	^*^0.490	0.487	0.492
Marital: Divorced/Widowed	-	-	-	-	-	-
Education: No education	^*^0.871	0.867	0.874	^*^0.238	0.235	0.242
Education: Primary	^*^0.924	0.921	0.928	^*^0.579	0.574	0.583
Education: Secondary	^*^1.035	1.031	1.038	^*^1.097	1.089	1.105
Education: Higher	-	-	-	-	-	-
Occupation: Civil servant/Police/ Army	^*^48.197	47.360	49.049	^*^12.578	12.317	12.845
Occupation: Private sector	^*^1.462	1.458	1.467	^*^1.300	1.290	1.311
Occupation: Entrepreneur	^*^0.777	0.775	0.779	^*^0.965	0.959	0.971
Occupation: Farmer/Fisherman/ Labor	^*^0.886	0.884	0.888	^*^0.693	0.689	0.698
Occupation: Others	-	-	-	-	-	-

*Note*: *AOR* Adjusted Odds Ratio, *CI* Confidence interval

^*^ *p* < 0.001

Meanwhile, the poorest female workers are possibly 0.254 times the richest to have other health insurance (AOR 0.254; 95% CI 0.252–0.256). The poorer female workers are 0.495 times less likely than the richest to have additional health insurance (AOR 0.495; 95% CI 0.492–0.498). Female workers with middle socioeconomic status are possibly 0.320 times less than the richest to have NHI (AOR 0.320; 95% 0.317–0.322). Furthermore, the richer female workers have 0.583 times less likely than the richest to have NHI (AOR 0.583; 95% CI 0.579–0.587).

Furthermore, all control variables also found a significant relationship with health insurance ownership. Female workers who live in urban areas are more likely to have NHI or other health insurance. All age groups have a lower probability than the ≥ 65 age group to have NHI based on age group. On the contrary, all age groups are likelier to have other health insurance than the ≥ 65 age group.

According to marital status, all groups are less likely than divorced/widowed to have NHI and other health insurance. Meanwhile, based on education level, no education and primary have a lower probability than higher education to have NHI and additional health insurance. On the other hand, secondary education has a lower likelihood than higher education of having NHI and other health insurance.

The last result, based on occupation type, civil servant/police/army, and private sector groups have a higher probability than other categories to have NHI and additional health insurance. On the other side, entrepreneurs, farmers/fishers/laborers are less likely to have NHI and other health insurance than different categories.

## Discussion

As one of the national development goals, health development aims to realize a healthy lifestyle for everyone and achieve the highest degree of public health. Law Number 40 concerning the National Social Security System (NSSS) in 2004 mandates that social security is something that must be available to all Indonesians, including the National Health Insurance (NHI) through the Social Security Administering Agency (SSAA) [31–33]. One of the obligations and roles of the government through SSAA Health is to provide the fulfillment of health rights to workers as part of the individual health efforts, which are integrated with the insurance payment scheme facilitated by SSAA Health [33].

SSAA Health data in August 2021 recorded the number of NHI participants reaching 225.96 million. The SSAA targets the National Health Insurance-Healthy Indonesia Card (NHI-HIC) program to get 88.51% of Indonesia's total population in 2022. This coverage is equivalent to the entire membership of 245.14 million people [34]. The study results found that only 71.3% of female workers became NHI participants. Referring to the SSAA target, the total membership coverage for female workers is still deficient. Massive efforts are needed to increase the ownership of health insurance in the worker segment, especially female workers, who biologically have more health risks than male workers due to childbirth.

The study shows that socioeconomic status is associated with health insurance ownership among female workers in Indonesia. The poor female workers (poorer and poorest) have less possibility of having NHI. This phenomenon is natural because a person's financial ability strongly correlates with health insurance ownership [35]. Every health insurance requires a contribution from all participants, so a person needs to have the income to pay a monthly fee when he wants health insurance [35]. A person in low socioeconomic conditions (poor and near-poor) will have limitations in buying health insurance. The research results in several countries also prove that financial ability significantly correlates with health insurance ownership [32, 36, 37].

Furthermore, all socioeconomic statuses of female workers have less possibility of having other health insurance. Several reasons can explain this phenomenon, including the insufficient knowledge of female workers on the importance of health insurance. This study's data analysis shows that most respondents (49.2%) are uneducated female workers. The results indicate no education and primary have a lower probability than higher education to have NHI and other health insurance. Several studies in various countries also prove that inadequate education is closely related to health insurance ownership [36–38]. In addition, from the health insurance provider perspective, the dissemination of health insurance products and benefits has not been done optimally to all segments of society. Hence, people are not well informed about the benefits and importance of having health insurance. Several other studies evidenced that low participation in health insurance is due to a lack of socialization [39, 40]. Based on a socioeconomic level, all groups generally have a better chance than the richest to have an NHI than other health insurance.

Female workers who live in urban areas are more likely to have NHI or other health insurance. Living in urban areas has a more significant potential to get broad information about everything, including the importance of having health insurance [41, 42]. In urban areas, access to information and health services is much higher than those in rural areas, so understanding people in urban areas about health insurance is better than those in rural areas. The better the understanding of health, the higher a person's tendency to have health insurance [43–45].

The study found that the ≥ 65 age group has the highest likelihood of having NHI. Many studies show that age has a close relationship with health insurance ownership. The older, the more health problems suffer, requiring more health services [37, 46, 47]. However, the AOR of the other insurance ownership decreases as the respondents get older. This condition is possible because the NHI benefits package is better than other insurances. On the other hand, NHI premiums also tend to be cheaper [48]. As health risks increase in the elderly, cheaper and more comprehensive options make more sense, even though they must be sacrificed the conveniences compared to other insurance [49].

The study also informs that all groups are less likely than divorced/widowed to have NHI and other health insurance based on marital status. Literature on health and mortality by marital status has consistently identified that unmarried individuals generally report poorer health and have a higher mortality risk than their married counterparts [7, 50, 51]. So they need more health insurance compared to those who are married. The observations in almost all countries show that families with low or poor economic status have more inadequate health conditions than families with moderate and reasonable economic levels [52–54].

Based on occupation type, the study shows civil servant/police/army has the highest probability of other categories of having NHI. This condition is more of an effect of regulations that include civil servants/police/army as mandatory participants in NHI. Premiums for these purposes are directly deducted from their salaries. While the private sector also has a greater possibility of participating in the NHI because the regulations require employers to bear the NHI premium for their workers [32, 49].

Discuss the social health insurance system in a country or NHI; we must understand that participation is mandatory and binding for the entire population. The principle of NHI is mandatory and cooperation between participants so that the target of NHI is universal health coverage so that the government can distribute the risk more broadly. Each country has a strategy and mechanism for achieving universal health coverage, and Indonesia implements a cross-subsidy policy in the NHI program. The government fully subsidizes the contribution from the state budget for the poor. In contrast, the government's contribution is partially supported (4%), and the rest is taken/deducted from their salary [55–57]. With a mechanism like this, the poor and government employees have a higher chance of having health insurance than other types of work or people with different socioeconomic statuses.

Based on the results, the government needs to be more active in involving employers to increase the ownership of health insurance in the context of the NHI. The main target is workers with a low socioeconomic.

### Study limitation

The study strengths analyzed big data as an analysis material to show generalized female workers' pictures up to the national level. Meanwhile, the study limitation utilized secondary data. The variables used are limited to those provided by the Ministry of Health of the Republic of Indonesia. Previous studies found other variables influencing health insurance ownership could not be investigated. These variables are cognitive capacity, prior commercial insurance ownership, having children, and family size [58–60].

## Conclusions

The study concluded that socioeconomic status affects health insurance ownership among female workers in Indonesia. Furthermore, all control variables also indicate a relationship with health insurance ownership among female workers: the type of residence, age group, marital status, education level, and occupation type.

Based on the results, the government needs to be more active in involving employers to increase the ownership of health insurance in the context of the NHI. The main target is workers with a low socioeconomy. On the other hand, the government needs to pay more attention to those who work in the informal sector. Their situation is more vulnerable, with incomes tending to be lower and uncertainty higher.

The government also needs to pay attention to female workers with middle wealth status. For the poorest female workers, the government has included them in the CAR mechanism. Meanwhile, female workers with moderate wealth must pay their own NHI premiums. When they are in good health, the contribution is not a problem, and it's different when their condition is sick; the amount of the NHI premium becomes heavy.

## Data Availability

The author cannot publicly share the data because a third party and authors who own the data do not have permission to share the data. The survey data set requested from the National Institute of Health Research and Development of the Indonesia Ministry of Health is available via https://www.litbang.kemkes.go.id/layanan-permintaan-data-riset/ for researchers who meet the criteria for access to confidential data.

## Abbreviations

NHI: National Health Insurance
NAR: Non-Contribution Assistance Recipient
CAR: Contribution Assistance Recipients
NSSS: National Social Security System
SSAA: Social Security Administering
NHI-HIC: National Health Insurance-Healthy Indonesia Card
AOR: Adjusted odds ratio
VIF: Variance Inflation Factor
